# The Development of the Innovation Readiness Inventory: An Assessment Tool to Assess Innovation Readiness of Nursing Organizations

**DOI:** 10.1177/23779608231202631

**Published:** 2023-09-21

**Authors:** Nina A. Lahti, Caroline Kevin, Sara Schulz, Katarina Meijers, Gerhard G. Bothma

**Affiliations:** 1 Centre for Innovation, 59562 Karolinska University Hospital, Stockholm, Sweden; 2 Emergency and Reparative Care, 59562 Karolinska University Hospital, Stockholm, Sweden; 3 InnoReadi, Stockholm, Sweden

**Keywords:** nurse-led innovation, innovation readiness, assessment tool, qualitative research, ‌design thinking, frontline innovation‌

## Abstract

Nursing organizations are expected to make contributions to innovation in Healthcare. However, they are often not innovation ready. How do nursing organizations know how *Innovation Ready* they are? What should nursing organizations do to become more *Innovation Ready*? In literature, there exists no consolidated resource to help nursing organizations to answer these questions. Therefore, this research aimed to support nurse-led innovation by developing an *Innovation Readiness Inventory* to be used as an assessment tool to qualitatively assess the innovation readiness of nursing organizations and support decision-making on actions to be implemented towards innovation readiness. The researchers performed a literature review to provide the theoretical basis for the assessment tool. Thereafter, the researchers engaged innovation experts and end-users in the form of nurse directors and managers, and frontline nurses. The researchers conducted semi-structured interviews, surveys, and design workshops and performed directed qualitative content analysis where relevant. Throughout, emphasis was placed on the scientific rigor of the research methodology with the intent to ensure the “trustworthiness” of the outcomes. To this end, the researchers implemented published best practices, when relevant and appropriate. As contribution to the discussion around nurse-led innovation, this research delivers the innovation readiness inventory that focusses the discussion on nursing organizations and the nurses within, and discusses challenges and opportunities related to the use thereof.

## Introduction

Nurses are well positioned to contribute to innovation in healthcare*.* Nurses have been acknowledged as the backbone of healthcare systems as they play a fundamental role in taking care of patients and providing round the clock services. In fact, it is estimated that as much as 80% of care within a healthcare system is provided by nurses (Baig et al., 2022). Nurses are natural innovators as they often introduce new approaches, ideas, and solutions to problems faced during the provision of care. Their understanding of patients, families, and communities provides a valuable perspective to the sustainability, scale-up, and use of innovations (Leary et al., 2022; Zuber & Weberg, 2020). Nurses have street credibility and trust relationships in an environment where this is paramount. They have extensive point-of-care experience and are usually mission driven to improve outcomes (Asch et al., 2014). Therefore, there is a need for healthcare systems to harness the innovation delivered by nurses.

Nurses are often absent as partners in healthcare innovation initiatives. While there are various reasons for this absence, the researchers believe that one of the main reasons is that nursing organizations are often not innovation ready. Asch et al. (2014) argue that successful system-wide innovation starts from within individual organizations and that direct efforts need to be implemented to adopt the supportive structures and processes required for innovation. In agreement with Asch et al. (2014), the researchers believe that, for nurses to actively take part in healthcare innovation outside of their organizations, they must focus on improving their innovation readiness from inside their organizations.

*How do nursing organizations know how innovation ready they are? What should nursing organizations do to become more innovation ready?* In literature, there exists no consolidated resource to help nursing organizations to answer these questions. Therefore, this research aims to support nurse-led innovation by developing an assessment tool to qualitatively assess the innovation readiness of nursing organizations and support decision-making on actions to be implemented towards innovation readiness.

## Methods

The research methodology encapsulated five main objectives:

1. Performing a literature review to develop the model for and prototype 1 of the Inventory (O1).
2. Engaging experienced innovation professionals to assess the construct of the Inventory (O2).
3. Engaging a specific nursing department within Karolinska University Hospital (KUH) to assess the practical operationalization thereof (O3).
4. Engaging frontline nurses to gain their perspective on hurdles for nurse-led innovation with the intent to assess whether the inventory addresses those hurdles (O4).
5. Considering all feedback in a combined analysis to deliver the innovation readiness inventory (O5).

**Figure 1.**
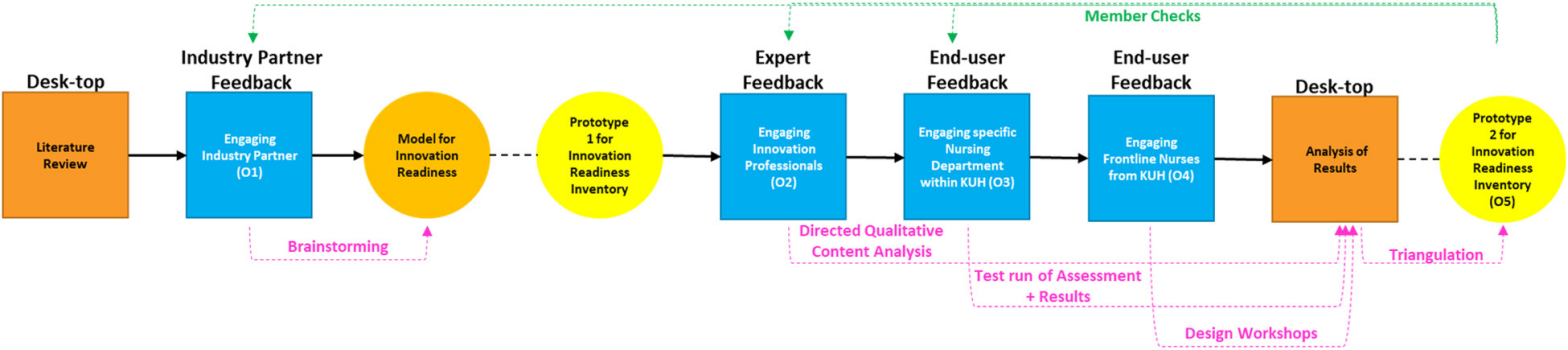
Summary of Process of Iteration and Validation.

Figure 1 aims to summarize the process of iteration and validation employed.

Following a literature review and a brainstorming session, a model for innovation readiness was developed from which the innovation readiness inventory was abstracted. Thereafter, interviews, surveys, and workshops were employed to elicit expert and end-user feedback, and appropriate qualitative research methodologies were employed to analyze the outcomes and incorporate it into an updated version of the innovation readiness inventory.

To meet research objectives 3–5, the researchers collaborated with the Emergency and Reparative Care Department within Karolinska University Hospital. The management structure of this department comprises 3 Nurse Directors (2 in Huddinge and 1 in Solna) and 36 Nurse Managers (23 in Huddinge and 13 in Solna). The total number of nurses and assistant nurses within this department is approximately 1,000 accounting for approximately 13% of the total number of nurses employed by Karolinska University Hospital.

## Results

### Literature Review

#### Frameworks and models for innovation readiness

Van den Hoed et al. (2022) recently performed a scoping review and identified four main factors and 10 sub-factors as contributing to innovation readiness in healthcare organizations.

Strategic course for innovation requires top management to prepare the organization`s long-term direction towards innovation readiness. It includes considering the role and importance of innovation in achieving the strategic objectives of the organization and allocates resources between current operations and innovation at the organizational level.

Climate for innovation focusses on creating a supportive organizational environment that contributes to innovation and focuses both at the team and organizational levels.

Leadership for innovation emphasizes the role of leadership provided by management and considers the attitude and behavior of upper managers in leading the way to become innovation ready as well as the role of middle managers to apply implementation policies and allocate required resources for innovation (Van den Hoed et al., 2022).

Commitment to innovation focusses on the individual employee with the objective of raising awareness of those behaviors and mindset required to enable a long-lasting commitment towards making individual contributions to innovation readiness.

As a framework, the review provided by Van den Hoed et al. (2022) represents a good point of departure. However, to support developing a deeper contextual understanding of concepts around innovation readiness, the researchers expanded the literature review to include additional models.

Flessa and Heubner (2021) performed a systematic literature review and analyzed three case studies to arrive at their model of adoption of a healthcare innovation.

The aim of their model is to answer the question of what must happen for innovation from outside an organization to become accepted innovation inside the organization and the model takes a systems approach in trying to meet this need (Flessa & Heubner, 2021). Flessa and Heubner (2021) highlight the role of the middle manager to act as “promoter” or champion for the implementation of innovation and that a leadership style that allows individuals the mental space to take risk, test, and experiment, and try and fail, will enhance their innovation behavior. They also emphasize that an innovation process that encapsulates idea generation and implementation requires professional and focused management, that is, proper planning, thorough organization, appropriate staffing, constant motivation, and many forms of control and synchronization. Flessa and Heubner (2021) advocate for the organization-wide communication and establishment of a definition of innovation and addressing the innovation culture and mindset at an organizational and individual level.

Turning attention to innovation frameworks focused on nursing organizations, Leary et al. (2022), described how schools of nursing can integrate innovation into their missions and position nurses as innovation leaders.

Leary et al. (2022) place heightened emphasis on external relationships and collaborations and the establishment of nurses as leaders in healthcare innovation. They emphasize the need for education in support of innovation leadership and methodologies and organizations providing individuals with the skills, tools, and resources to innovate.

Stacy Kriel (2021) introduced the model for clinical inquiry, of which innovation is one of four key components, that has been adopted by *Penrose St. Francis Health Services* and specific steps have been taken to operationalize this model.

*Penrose St. Francis Health Services* established a centralized multi-disciplinary council, supported by an executive sponsor, to advance interdisciplinary collaboration and to transform care through the establishment of a culture of inquiry and innovation. The council accomplishes this by providing an environment for sharing and discussing ideas and lessons learned, facilitating dissemination of findings, and providing continuing educational programs around innovation.

Zuber and Weberg (2020) emphasize than frontline nurses need to be intentionally engaged for successful innovation to occur and that innovation requires the same rigor and attention than other change management and improvement efforts. They further the discussion around innovation readiness by introducing the concept of an “Innovation Microclimate,” where formal or informal leaders act as a bridge between an early state of individual creativity and organizational innovation as an interim state towards a more mature organizational culture of innovation.

### Definition of Nurse-led Innovation

Sanford (2019) and Flessa and Heubner (2021) differentiate between an invention and an innovation. While an invention is a process, product, or procedure that was created for the first time, innovation not only encapsulates the process of improvement thereof, but also taking steps towards implementation, acquiring new knowledge along the way.

While a definition of innovation can simply be “solving pressing problems (Leary et al. (2022), Flessa and Heubner (2021), and Joseph et al. (2019)” believe that a definition for nurse-led innovation needs to be specific and tailored to the needs of the organization. Joseph et al. (2019) provide examples of the types of innovations in nursing that include creating care delivery models, transforming processes to improve care, developing patient care interventions, and advancing research and translational methods, facilitating communication and collaboration, harnessing technology and data, enabling role transitions and developing teaching methods.

### Development of Innovation Readiness Model and Prototype 1 of the Innovation Readiness Inventory (O1)

**Figure 2.**
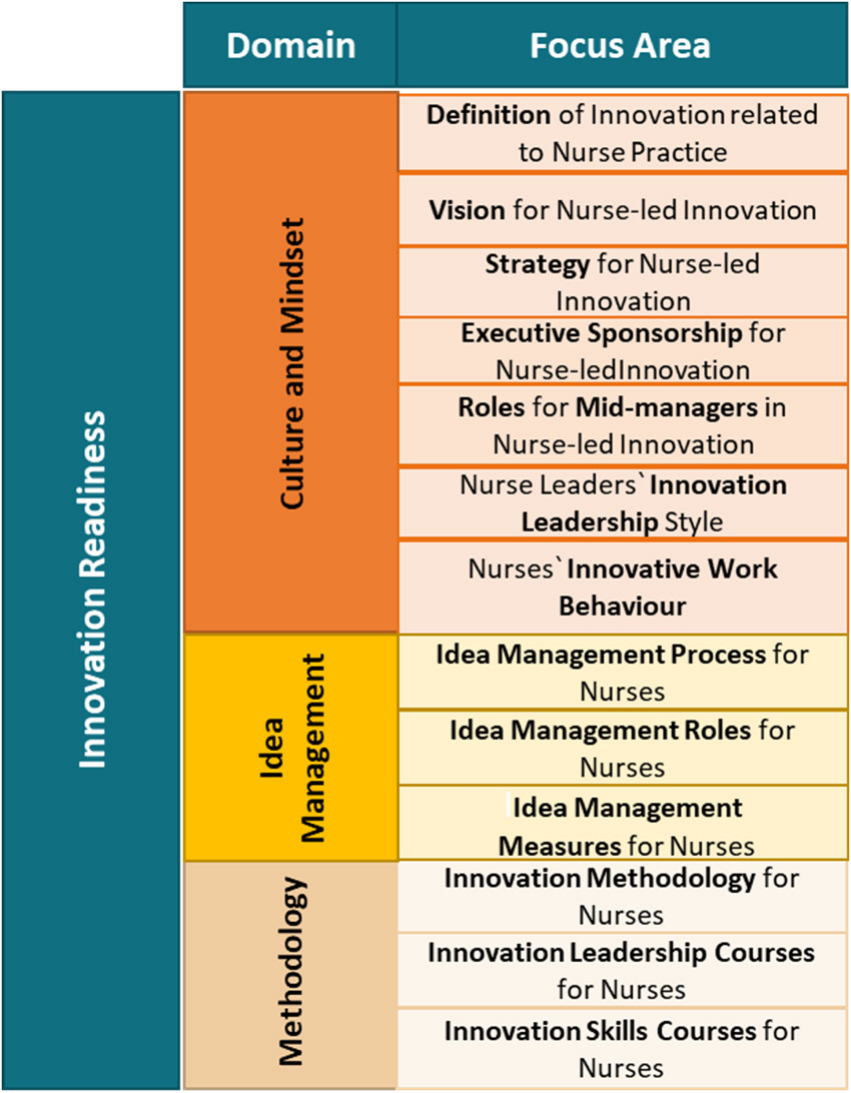
Evidence-based innovation readiness model for nurse-led innovation.

From the literature review, the researchers developed an innovation readiness model for nurse-led innovation (Figure 2).

**Figure 3.**
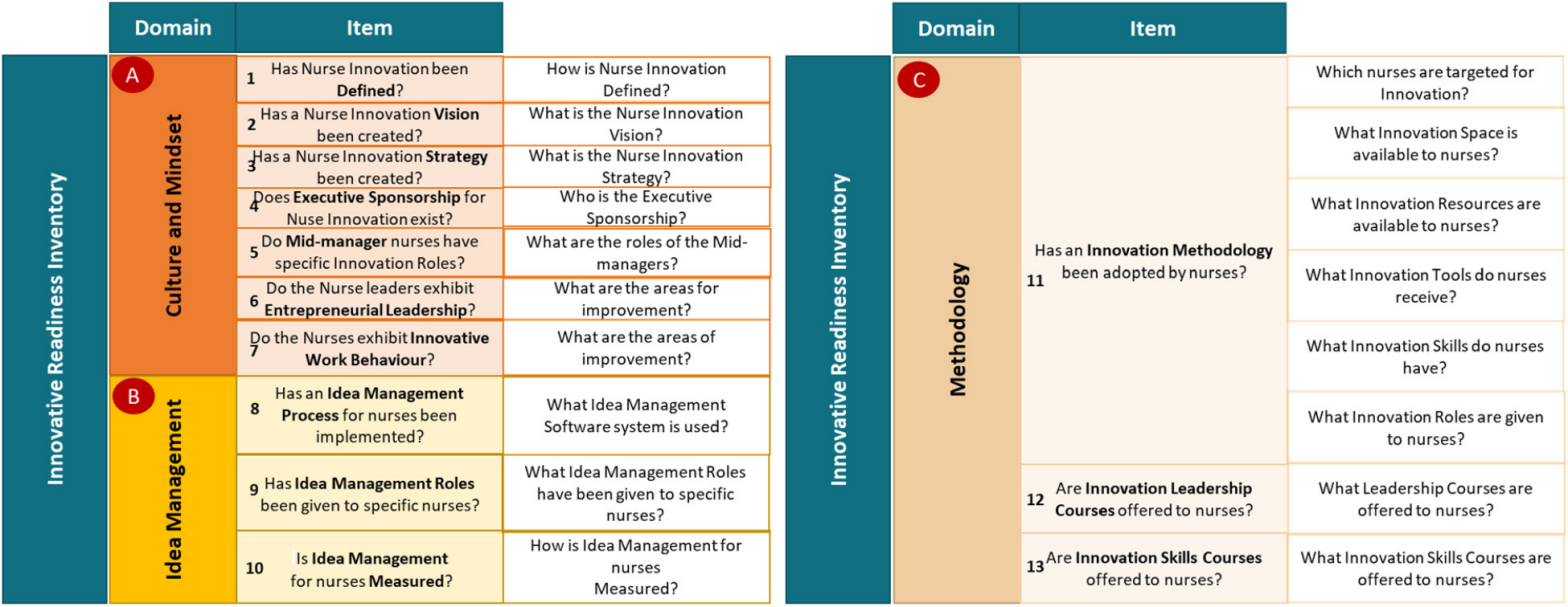
Prototype 1 for innovation readiness inventory.

Note that the decision was taken to call central themes “Domains” and aspects “Focus Areas.” Using the innovation readiness model as basis, the innovation readiness inventory (Figure 3) was abstracted to be assessed as a prototype for the assessment of innovation readiness of nursing organizations.

In the inventory (Figure 3) and as compared to the model (Figure 2), each of the “Domains” was assigned an alphabet letter (A, B and C), “Focus Areas” were replaced with “Item,” and each “Item” were assigned a number (1–13). Also, statements constituting each “Focus Area” in the model were replaced with closed-ended questions in the inventory and follow-up questions.

### Assessment of the Construct of the Innovation Readiness Inventory (O2)

The researchers interviewed 12 Innovation Professionals with cumulative years of experience in healthcare innovation of more than 170 years.

When conducting directed qualitative content analysis, the researchers considered the manifest content, and the unit of analysis was phrases expressing “Agreement or similar,” “Disagreement or similar” and “Re-phrasing or similar,” and coding was done manually. A formative categorization matrix was developed and applied, and the results indicated that the Innovation Professionals agreed that the construct of the Inventory was comprehensive.

“It is really well executed and laid out.” – Interviewee 1

“Great tool for understanding where we are and what to do next.” – Interviewee 2

“Like the Heat Map. Makes things visible and supports communication around it.” – Interviewee 7

“Broad tool that covers a lot around innovation. It is logical and easy to understand.” – Interviewee 9

The following general concerns were voiced:

1. The inventory should not be seen as another task to be completed by nurses, but it should contribute to an assessment of the overarching innovation mindset and support an increase in the innovation awareness and confidence among nurses.
2. The inventory should not position innovation as a list of actions but should rather be used to support the development of an understanding of the overarching leadership and actions required.
3. “Items” should not be generalized to apply equally across all nurses within the nursing organization. For example, from “Has an Innovation Methodology been adopted among nurses?” It should not be understood that all nurses need to have all tools and skills related to the methodology to the same extent.
4. Structures and processes encapsulated by the inventory should emphasize “Fit-for-Purpose” for a particular nursing organization, as this would support an understanding that there is not a one-size-fits-all intent behind the Inventory.

With reference to Figure 3, Table 1 indicates the “Items to be re-phrased” and “Items to be added” emerging from the directed qualitative content analysis.

**Table 1.** “Items to be Re-Phrased and Added” Resulting from Directed QCA.

Domain	Items to be re-phrased	Items to be added
**Culture and mindset**	Add “and Communicated” to Items 1–3.	Availability and access to innovation support functions
	Add “formal or informal” to Item 5.	Structures and processes for collaboration across departments and disciplines
	Replace “Entrepreneurial” with “Innovation” in Item 6.	Existence of rewards and recognition
**Idea management**	Add “Fit-for-purpose” to Items 8–10.	
**Methodology**	Add “Fit-for-purpose “to Item 11.	
	Replace “Courses” with “Development Program” in Item 12.	
	Add “Fit-for-purpose” to Item 13.	

### Assessment of the Practical Operationalization of the Innovation Readiness Inventory (O3)

#### Using the innovation readiness inventory (prototype 1) as assessment tool

**Figure 4.**
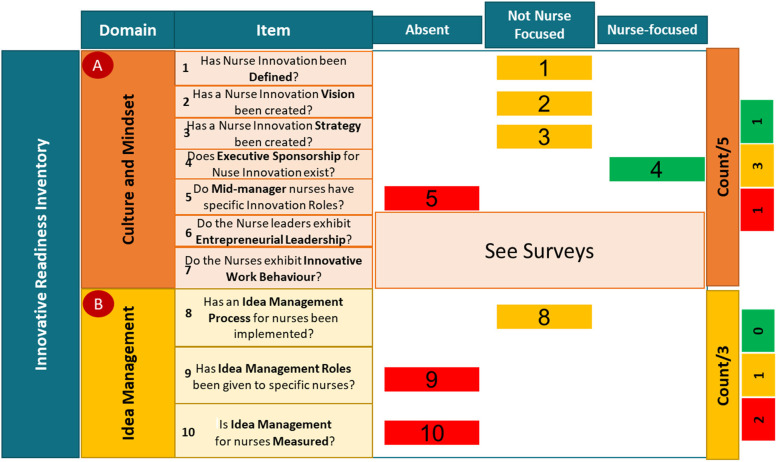

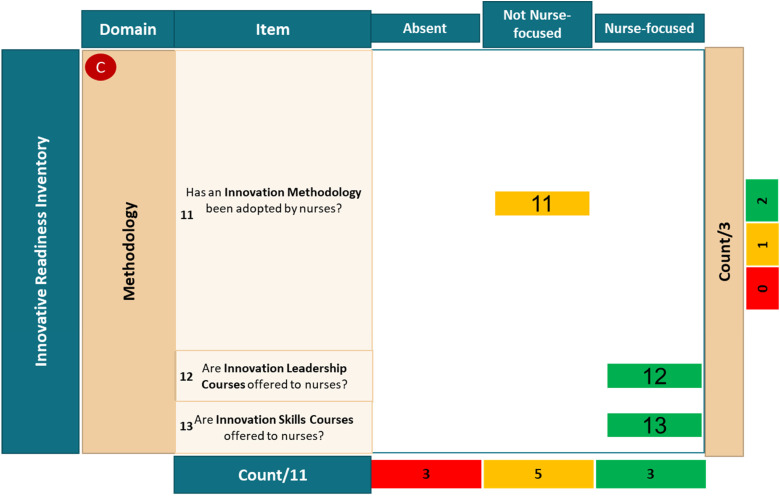
Qualitative assessment of the innovation readiness of the nursing organization within the emergency and reparative medicine department.

Interviews with the nurse directors were reviewed, and the outcomes were used to make a qualitative assessment of the innovation readiness of the department. Figure 4 aims to show how the outcomes of the use of the Inventory were communicated.

Thus, in eight out of a possible eleven “Items,” the “Item” was either absent or not nurse—focused within the department, with “Culture and Mindset” and “Idea Management” possible candidates for improvement.

#### Using surveys to support the innovation readiness inventory (prototype 1) as assessment tool

Three surveys were employed, that is., the *Innovation Behaviour Survey,* the *Innovation Support Survey* and the *ENTRELEAD Survey*. Responses from nurse directors (100% response rate) and nurse managers (81% response rate) were analyzed and visualized to aid describing the outcomes.

Analysis of the *Innovation Behaviour Survey* showed that the nurse managers indicated creating opportunities to interact with other departments as the biggest immediate need for improving their innovative work behavior.

Analysis of the *Innovation Support Survey* showed that the nurse managers indicated that the nurse directors could enhance their innovation support by:

1. Showing more appreciation for good ideas.
2. Supporting Nurse Managers more to implement ideas.
3. Being more tolerant of mistakes and errors during implementation of ideas.

Analysis of the *ENTRELEAD Survey* showed that the nurse managers indicated that the nurse directors could enhance their entrepreneurial leadership by more clearly communicating their vision for the department.

#### Determining the perceived value of the outcomes of the assessment

**Figure 5.**
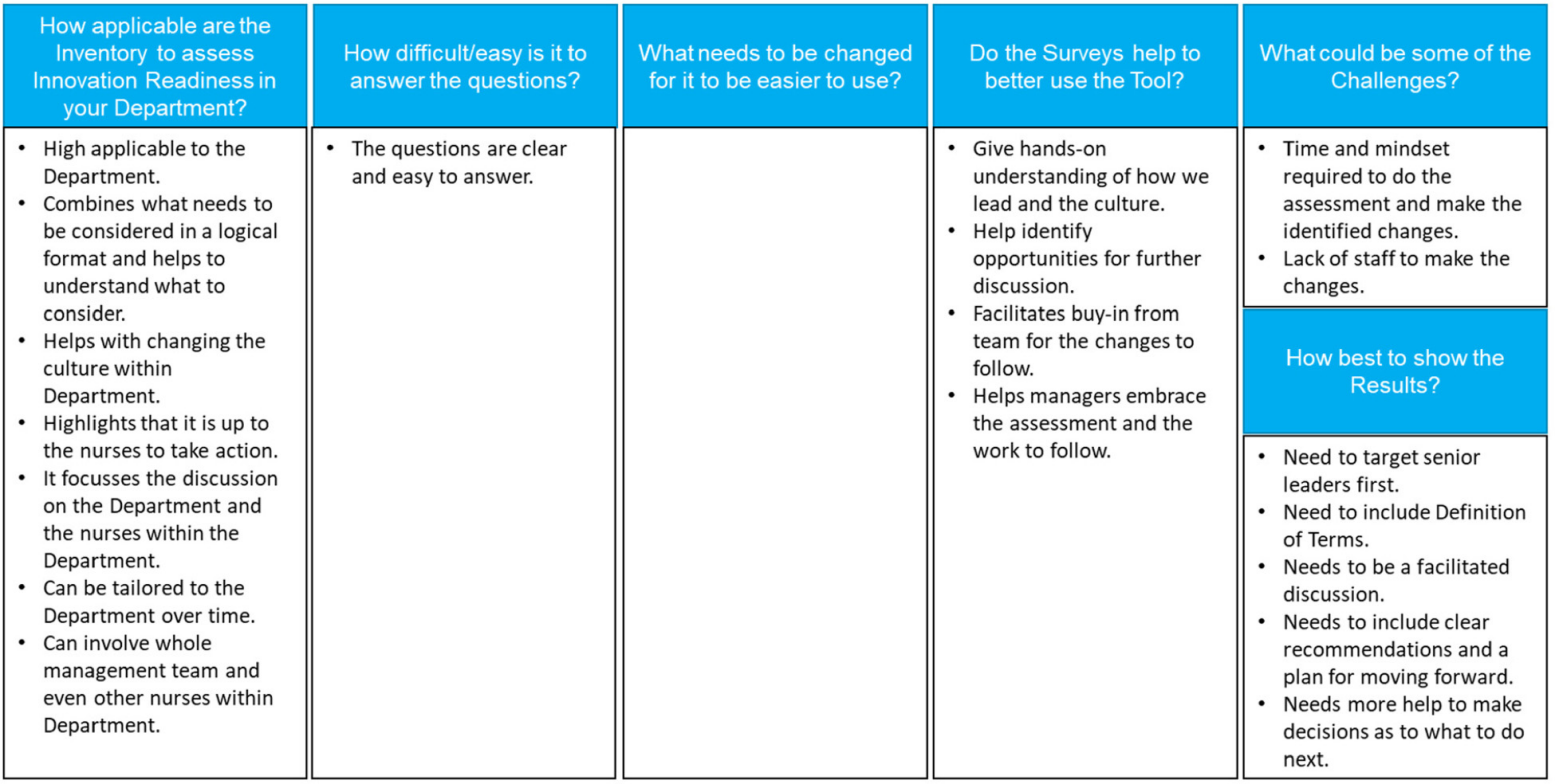
Comments received from nurse directors related to operationalization of the inventory.

During a 2^nd^ round of interviews with the nurse directors, they were asked to comment on the use of the inventory, the need of the surveys and the value of the outcomes, and their comments are summarized in Figure 5.

Thus, the nurse directors agreed that the inventory is applicable to assess the innovation readiness of their department, is easy to use and does not require changes, and that the surveys added significant value to the overall assessment. However, they also voiced some challenges, most notably that they would require more support as to what to do after the assessment.

“This can help me Change the Culture for Innovation.” – Nurse Director 1

“It is not about anyone but the Nurse.” – Nurse Director 2

“*I think it is really good and it really got me thinking.”* – Nurse Director 3

### Assessment of Whether the Innovation Readiness Inventory Addresses the Hurdles Identified by Frontline Nurses (O4)

Three workshops were attended by 15 Frontline Nurses.

In Workshop 1, participants highlighted “Submitted ideas do not get feedback,” “Ideas have nowhere to go,” and “Ideas are not shared between Departments” as main challenges and agreed on “Culture and Mindset,” “Idea Management,” “Innovation Tools/Skills,” and “Collaboration across Departments” as themes to take to Workshop 2.

In Workshop 2 participants refined and specified what they would like to focus on:

1. “Management must embrace innovation and development”.
2. “An easier process for submitting and tracking ideas”.
3. “More time for nurses to be innovative”.
4. “An innovation mindset and behaviour among all nurses”.

In Workshop 3 participants were asked to use “How Might We” questions and brainwriting to generate ideas. The ideas generated were

1. Creating communication opportunities with management where nurses share evidence and examples about their ideas to get management to support the nurses in implementing those Ideas.
2. Creating a simple system that focusses on gathering ideas on, for example, possibilities for new ways of working, and that tracks the progress of an idea with feedback provided throughout.
3. Scheduling regular meetings across departments and disciplines, where ideas are shared and opportunities are identified to implement some ideas from other departments.
4. Asking management for the time and mandate to create a nurse-led separate team that focus on building nurse competencies and culture related to innovation from within the nursing organization.

### Update of the Innovation Readiness Inventory

**Figure 6.**
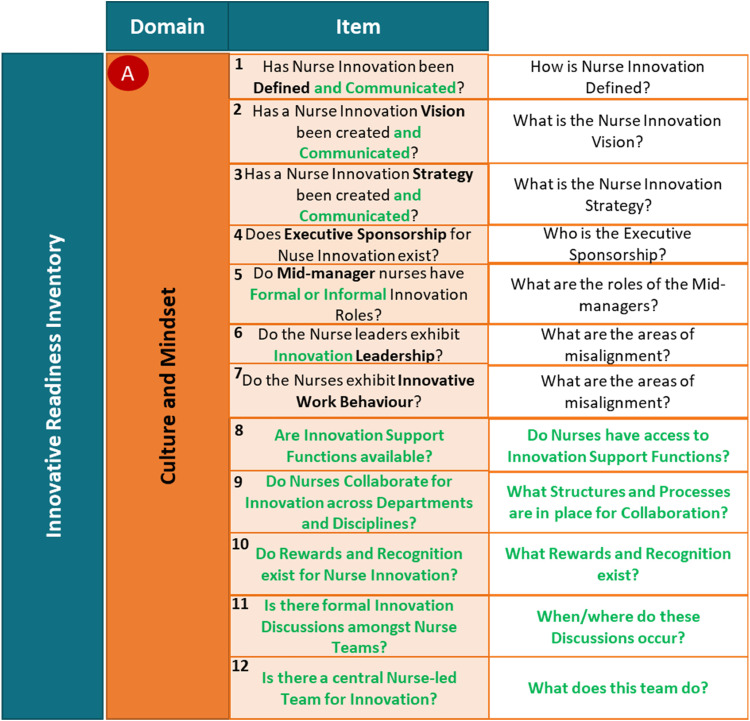

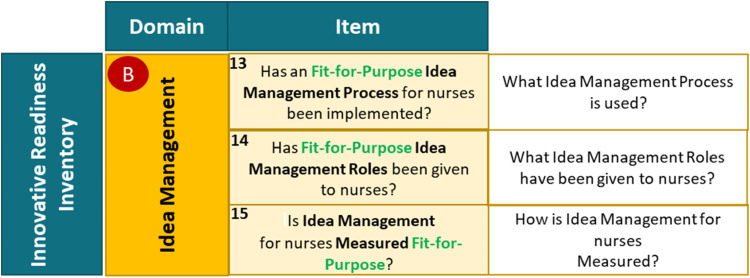

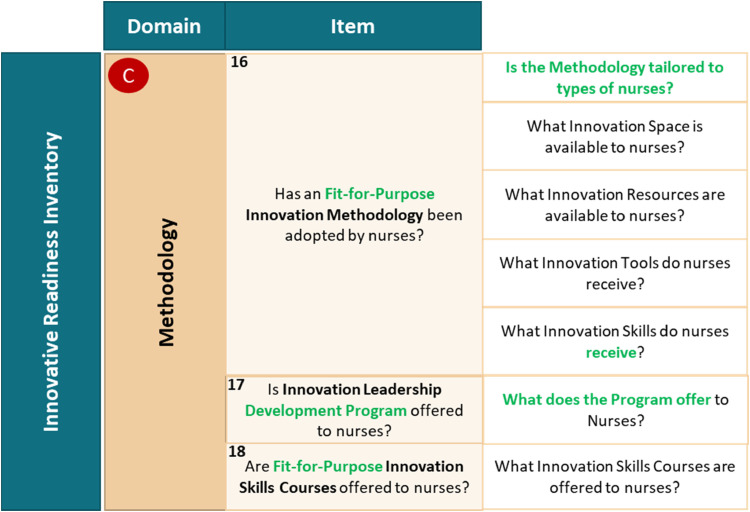
The innovation readiness inventory.

The researchers used triangulation, considering all results, to update prototype 1 to arrive at the Innovation Readiness Inventory for Nursing Organizations (Figure 6). Updates are highlighted in “green.”

Triangulation also highlighted the following considerations around the use of the Inventory as an assessment tool:

1. Need to target the right audience.
2. Need to understand the specific organizational context it is to be applied within.
3. Need to provide definitions of terminology used.
4. Need to be used together with a facilitated discussion, also when results are communicated.
5. Need to be appropriate supplemented to facilitate better decision-making as to what to do next.
6. Need to consider how to benchmark.
7. Might be considered to contain too many “Items.”

## Discussion

The results indicated that the “Domains” and “Items” contained within prototype 1 of the Inventory (Figure 3) were deemed relevant, and therefore, none were omitted from the updated innovation readiness inventory (Figure 6). However, 11 “Items” required re-phrasing, while 5 new “Items” were added to the inventory.

Availability and access to innovation support functions (Item 8) was suggested by 6 out of 12 interviewees and receives support in literature (Leary et al., 2022). Collaboration across departments and disciplines (Item 9) was suggested by 2 out of 12 interviewees, the nurse managers, and the frontline nurses and is also supported in literature (Leary et al., 2022; ÒHara et al., 2022). Rewards and recognition for nurse innovation (Item 10) was suggested by 5 out of 12 Interviewees and receives support from authors like Van den Hoed et al. (2022) and ÒHara et al. (2022). Formal innovation discussions among nurse teams (Item 11) were suggested by the nurse managers and the frontline nurses and Leary et al. (2022) and ÒHara et al. (2022) support this suggestion. Lastly, a nurse-led central team for innovation (Item 12) was suggested by the frontline nurses and is supported in literature by authors like Van den Hoed et al. (2022), ÒHara et al. (2022), and Kriel (2021).

Items 1, 2, and 3 were re-phrased by the addition of “and Communicated” as a general suggestion across the participant groups. The requirement of clear communication of the innovation definition, vision, and strategy is consistently highlighted in literature, including in the framework and models considered in the literature review. This also holds true for the re-phrasing of Items 13, 14, 15, 16, and 18 to include “Fit-for-Purpose.” Three out of 12 interviewees suggested the re-phrasing of Item 5 to include “Formal or Informal,” a suggestion supported by Zuber and Weberg (2022) and Luz et al. (2021). Six out of 12 interviewees suggested the replacement of “Entrepreneurial” in Item 6 with “Innovation” as it was argued that different nursing organization would require different types of leadership styles to facilitate the Innovative Work Behaviour of the nurses. Lastly, one out of 12 Interviewee suggested the re-phrasing of Item 17 to include “Development Program” in the place of “Course,” a suggestion supported by Van den Hoed et al. (2022), ÒHara et al. (2022), and Leary et al. (2022).

One of the main barriers to overcome related to nurse-led innovation is the nurses` view of innovation as another task. The nurse directors highlighted time as a barrier to performing the innovation readiness assessment and implement the changes, while the frontline nurses identified “More time for nurses to be innovative” as a hurdle. Therefore, there is a risk that the use of the inventory can be viewed as another task to be performed by nurses and, more importantly, that the “checklist” nature of the inventory can lead to a misunderstanding of innovation being a list of activities, taking the focus from innovation culture, mindset and leadership.

Four out of 12 interviewees suggested the need for a willingness to change towards innovation readiness as an overarching requirement for, not just the successful use of the inventory as an assessment tool, but also for the willingness to act after the assessment. Nilsen et al. (2020) highlight that the successful implementation of change among frontline nurses in Sweden required those nurses to have an opportunity to influence the change, being prepared for the change as well as valuing the changes to be made as “benefitting themselves and/or their patients,” that is, fit-for-purpose. The researchers have previously highlighted the inclusion of “fit-for-purpose” in relevant “Items” within the Inventory. The researchers have also highlighted the comment from the nurse directors that the use of the surveys as part of the assessment supported achieving buy-in from the nurse managers for the assessment and the changes to come. Thus, the surveys used as part of the assessment support change management around innovation.

Two out of 12 interviewees suggested that the Inventory might contain too many “Items,” a challenge augmented by the addition of five new “Items.” While further testing of the operationalization of the inventory beyond the targeted department will shed more light on this potential challenge, targeting the senior leader/s through facilitated discussions, applying the specific organization context, and the “fit-for-purpose” intent, will disqualify non-relevant “Items” early on in the assessment process.

### Strengths and Limitations

Prototype 1 of the innovation readiness inventory was built on a strong evidence-based that facilitated the research methodology used and ensured the quality of the interactions with the participants during the interviews and workshops. The researchers interviewed experienced innovation leaders from Sweden, USA, and Australia active within different sectors of the healthcare industry and directly engaged the frontline nurses as, in many ways, they are the “consumers” of the actions that are implemented as a result of the assessment of innovation readiness. Throughout the focus was on the application of scientific rigor during the planning, execution, and reporting of the research, thereby enhancing the external validity thereof.

However, opportunities for improvement and continued research exist and the researchers` Recommendations in this regard are (In suggested order):

1. Assessing the ability of the inventory to drive the implementation of measurable change towards innovation readiness.
2. Extending the use of the inventory across whole nursing organizations within the same care setting.
3. Developing a digitalized toolkit and testing it within the context of a typical nursing organization.
4. Extending the use of the inventory between nursing organizations of different care settings.
5. Enhancing the capabilities of the inventory to allow for intra- and inter-organizational benchmarking.

### Implications for Practice

The Innovation readiness Inventory provides nursing organization with a tool with which to qualitatively access the level of innovation readiness at an organizational level, while also providing insight into those actions to take to improve the innovation readiness.

## Conclusion

The research adds to the discussion around nurse-led innovation by delivering the innovation readiness inventory as assessment tool that can be used to assess the innovation readiness of nursing organizations and support decision-making as to what to focus on to increase innovation readiness. When using the innovation readiness inventory, care must be taken not to position it as a checklist and another task to be performed. Rather, it should be viewed as tool towards understanding those possible actions required to move towards the establishment of on innovation culture and mindset among nurses.

## Supplemental Material

sj-docx-1-son-10.1177_23779608231202631 - Supplemental material for The Development of the Innovation Readiness Inventory: An Assessment Tool to Assess Innovation Readiness of Nursing Organizations Click here for additional data file. Supplemental material, sj-docx-1-son-10.1177_23779608231202631 for The Development of the Innovation Readiness Inventory: An Assessment Tool to Assess Innovation Readiness of Nursing Organizations by Nina A. Lahti, Caroline Kevin, Sara Schulz, Katarina Meijers and Gerhard G. Bothma in SAGE Open Nursing
