# Supplementary material for: The Development of the Innovation Readiness Inventory: An Assessment Tool to Assess Innovation Readiness of Nursing Organizations
Source: SAGE Open Nurs. 2023 Sep 21;9:23779608231202631. doi: 10.1177/23779608231202631 (PMC10517619; doi:10.1177/23779608231202631)
Supplement: sj-docx-1-son-10.1177_23779608231202631 - Supplemental material for The Development of the Innovation Readiness Inventory: An Assessment Tool to Assess Innovation Readiness of Nursing Organizations [file sj-docx-1-son-10.1177_23779608231202631.docx]

# Supplementary Material

**Supplement 1**

**Validation Form**

**Innovation Readiness Model**


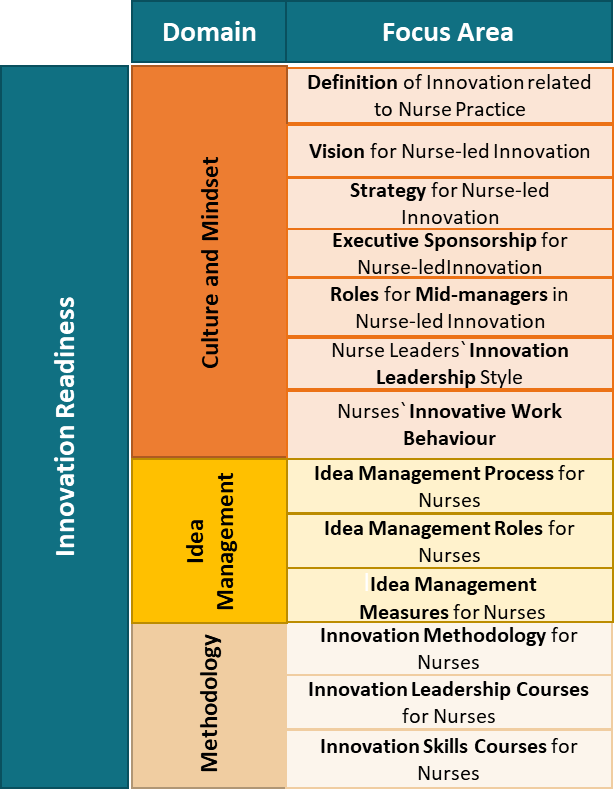


**Considering the Innovation Readiness Model:**

- Are there *Domains* and/or *Focus Areas* that are not relevant and, therefore, should be *removed*.
- Are there *Domains* and/or *Focus Areas* that have not been considered and should be

*added*.

- What opportunities do you see when applying this model to the Innovation Readiness of nursing organizations.
- What challenges do you see when applying this model to the Innovation Readiness of nursing organizations.

**Innovation Readiness Inventory**


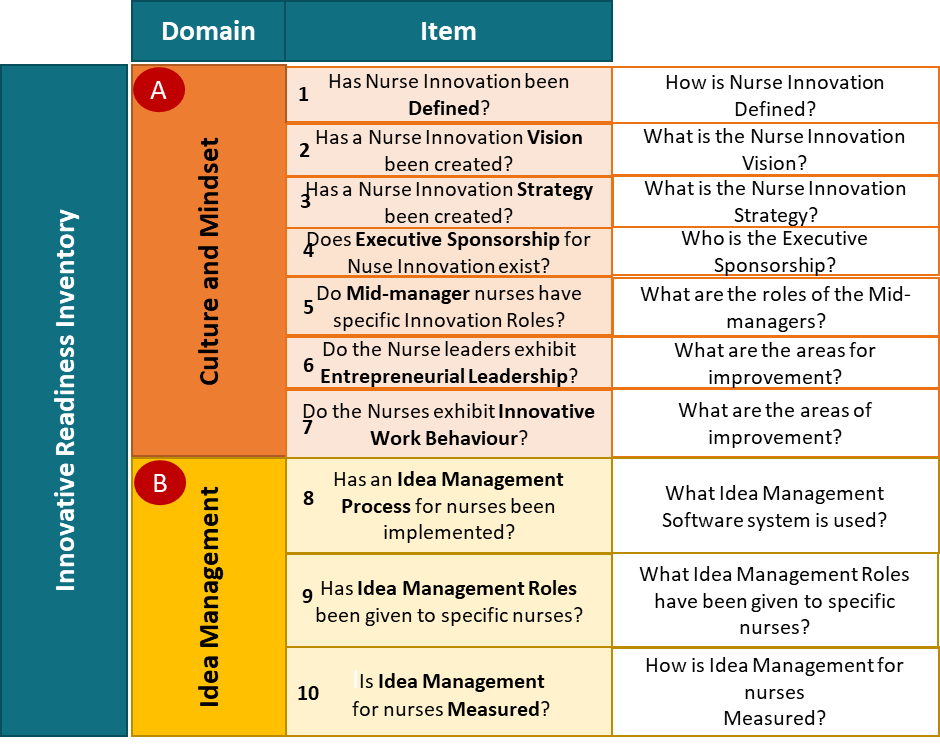


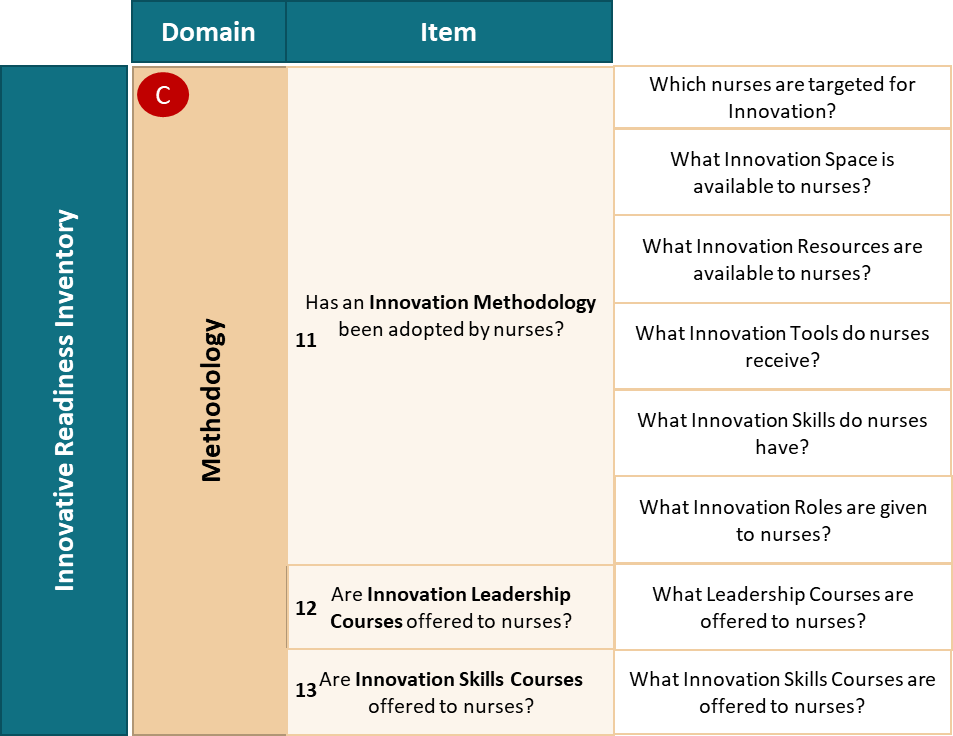


**Considering the Innovation Readiness Inventory:**

- Are there any Questions that are not relevant and, therefore, should be removed.
- Are there Questions that have not been considered and should be added.
- Would you reframe certain Questions? How would you reframe them?
- What opportunities do you see with employing this inventory when assessing the Innovation Readiness of nursing organizations.
- What challenges do you see with employing this inventory when assessing the Innovation Readiness of nursing organizations.

**Assessing Key Statements**

Considering each of the following statements, do you support them? What would you change in the statement to support them?

**Statement 1:** A clear and discipline-specific definition of nursing innovation is foundational to the *Innovation Readiness Model* and, as such, should be included as Focus Area into the model.

**Statement 2:** A transformational leadership style, as enhanced by an entrepreneurial leadership style (entrepreneurial orientation), facilitates the innovative work behaviour (IWB) of nurses. Therefore, it is included into the *Innovation Readiness Model* as a Focus Area.

**Statement 3:** Nursing organizations needs to assign specific roles within the innovation process to mid-managers, including idea management (a), and they should capitalize on the championship roles these managers could play by creating an innovation climate within which innovation champions play a recognizable role towards innovation success (b).

Therefore, considering the role of the mid-managers in nurse innovation is included as Focus Area into the *Innovation Readiness Model*.

**Statement 4:** The extent to which nurses in a nursing organization exhibit innovative work behaviour (IWB) largely determines the success of nurse innovation (a) and that nursing organizations need to create the culture and mindset and adopt the required leadership styles to facilitate it (b). Therefore, Nurses` Innovative Work Behaviour is included as a Focus Areas within the *Innovation Readiness Model*.

**Statement 5:** Successful innovation management requires the adoption of an Idea Management System (IMS) that are fit-for-purpose for a nurse organization and targets the front-line nurse (a), and that nurses and their managers need to be assigned specific roles with the idea management process (b). Therefore, Idea Management is included as a Domain within the *Innovation Readiness Model*, with Focus Areas dedicated to the process, roles of nurses and the measurement thereof.

**Statement 6:** Successful innovation by nurses requires the adoption of an innovation methodology across the whole organization that fit the needs of and facilitate the innovation process (a). Specifically, nurse organizations can consider the adoption of design thinking as an innovation approach and provide the necessary support for it to be used successful by all nurses (b). Therefore, Innovation Methodology is included both as a

Domain and a Focus Area within the *Innovation Readiness Model*, together with Focus Areas around educational support.

# Supplement 2

**Innovation Readiness Inventory Questionnaire**

**General Questions to Person being Interviewed**

What is the *Title of your Role*?

Please provide a short *Description of your Role*?

What are the *Total Years of your Professional Experience*? How long (years) have you been *Active within Innovation*?

**Culture and Mindset**

**Question 1:** Has Nurse Innovation been *Defined*? How is Nurse Innovation *Defined*?

**Question 2:** Has a Nurse Innovation *Vision* been created? What is the Nurse Innovation

*Vision*?

**Question 3:** Has a Nurse Innovation *Strategy* been created? What is the Nurse Innovation

*Strategy*?

**Question 4:** Does *Executive Sponsorship* for Nurse Innovation exist? Who is the Executive Sponsor?

**Question 5:** Do *Mid-manager* nurses have specific *Innovation Roles*? What are the *Roles of Mid-managers*?

**Question 6:** Do the *Nurse leaders* exhibit *Entrepreneurial Leadership*? What are the areas for improvement?

**Question 7:** Do the *Nurses* exhibit *Innovative Work Behaviou*r? What are the areas for improvement?

**Idea Management**

**Question 8:** Has an *Idea Management Process* for *Nurses* been Implemented? What *Idea Management Software* system is used?

**Question 9:** Have *Idea Management Roles* been given to specific *Nurses*? What *Idea Management Roles* have been given to specific *Nurses*?

**Question 10:** Is *Idea Management* for *Nurses Measured*? How is *Idea Management* for

*Nurses Measured*?

**Question 11:** Has an *Innovation Methodology* been adopted by *Nurses*?

- Which Nurses are *Targeted* for Innovation?
- What Innovation *Space* is available for Nurses?
- What Innovation *Resources* are available to Nurses?
- What Innovation *Tools* do Nurses receive?
- What Innovation *Skills* do Nurses have?
- What Innovation *Roles* are given to Nurses?

**Question 12:** Are *Innovation Leadership Courses* offered to *Nurses*? What *Leadership Courses* are offered to *Nurses*?

**Question 13:** Are *Innovation Skills Courses* offered to Nurses? What *Innovation Skills Courses* are offered to Nurses?
